# Tomato mitogen-activated protein kinase: mechanisms of adaptation in response to biotic and abiotic stresses

**DOI:** 10.3389/fpls.2025.1533248

**Published:** 2025-02-03

**Authors:** Yumei Shi, Zhifang Zhang, Zhenghao Yan, Honglong Chu, Changxin Luo

**Affiliations:** College of Biological and Food Engineering, Qujing Normal University, Qujing, Yunnan, China

**Keywords:** mitogen-activated protein kinases, biotic stress, abiotic stress, signal transduction, tomato

## Abstract

Plants live under various biotic and abiotic stress conditions, and to cope with the adversity and severity of these conditions, they have developed well-established resistance mechanisms. These mechanisms begin with the perception of stimuli, followed by molecular, biochemical, and physiological adaptive measures. Tomato (*Solanum lycopersicum*) is a globally significant vegetable crop that experiences several biotic and abiotic stress events that can adversely impact its quality and production. Mitogen-activated protein kinases (MAPKs) in tomato plants have crucial functions of mediating responses to environmental cues, internal signals, defense mechanisms, cellular processes, and plant development and growth. MAPK cascades respond to various environmental stress factors by modulating associated gene expression, influencing plant hormone synthesis, and facilitating interactions with other environmental stressors. Here, we review the evolutionary relationships of 16 tomato SlMAPK family members and emphasize on recent studies describing the regulatory functions of tomato SlMAPKs in both abiotic and biotic stress conditions. This review could enhance our comprehension of the MAPK regulatory network in biotic and abiotic stress conditions and provide theoretical support for breeding tomatoes with agronomic traits of excellent stress resistance.

## 1 Introduction

Tomato (*Solanum lycopersicum*) is a crucial vegetable crop globally. Tomato cultivation is a major industry, and global production was estimated at 182 million tons in 2018, rising to 186 million tons in 2020 (Collins et al., 2022). Nonetheless, several abiotic and biotic factors affect tomato cultivation. Drought, salinity, extreme temperatures, and nutrient deficiencies are abiotic stress factors, while biotic stress originates from insects, fungi, bacteria, nematodes, and viruses (Kissoudis et al., 2015; Collins et al., 2022). Global climate change has led to harsh field environments, resulting in consistently increasing agricultural losses and diminishing production of gains. Numerous climate models have predicted high frequency of extreme temperatures, floods, and droughts in the future (Boyer et al., 2013; Chen et al., 2021). Various stimulus response mechanisms and activation strategies have emerged in plants to meet these challenges. Plant receptors are a key element in plant-environment interaction, as they transmit information and enable the plant to recognize its surroundings (Osakabe et al., 2013; Dievart et al., 2020). Mitogen-activated protein kinase (MAPK) cascades are critical elements of all eukaryotic signaling networks. The MAPKs function downstream of sensors and receptors and facilitate cellular responses, thereby achieving integrated plant immunity, plant development and growth, and adaptation to an ever-changing environment (Zhang and Zhang, 2022). The MAPK cascade pathway includes at least one MAPK, mitogen-activated protein kinase kinases (MAP2Ks, MKKs, and MAPKKs), and mitogen-activated protein kinase kinase kinases (MKKKs, MAPKKKs, MAP3Ks, and MEKKs) (Tena et al., 2001). The abovementioned three kinase tiers in a cell comprise numerous members that function to ensure that the transmitted signal has the required specificity (Zhang and Klessig, 2001). Plant MAPK cascades are crucial in governing plant growth and facilitating plants’ response to an array of stress stimuli, such as pathogen invasion, injury, temperature fluctuations, salinity, ultraviolet (UV) exposure, osmotic changes, reactive oxygen species (ROS), drought, and ozone (Meng and Zhang, 2013; Lin et al., 2021; Majeed et al., 2023). Here, we present a review of recent advances in research on MAPK cascades implicated in signaling networks in tomato for coping with abiotic and biotic stress conditions.

## 2 Overview of MAPKs

Plant MAPKs, as a crucial component of intricate signaling networks, play vital roles in enabling plants to recognize and interact with environmental cues and internal signals; they also mediate defense mechanisms, regulate growth and development, and fine-tune various cellular processes (Colcombet and Hirt, 2008; Lin et al., 2021). MAPKs are activated by the dual phosphorylation of Thr and Tyr residues in a TXY motif located in the activation loop between subdomains VII and VIII by their upstream MAPK kinases. Subsequently, MAPKs phosphorylate their substrates, which are mainly transcription factors. These transcription factors then trigger downstream reactions (Hazzalin and Mahadevan, 2002; Wu et al., 2007).

### 2.1 MAPK cascades and their influence on signal transduction

**Figure 1:**
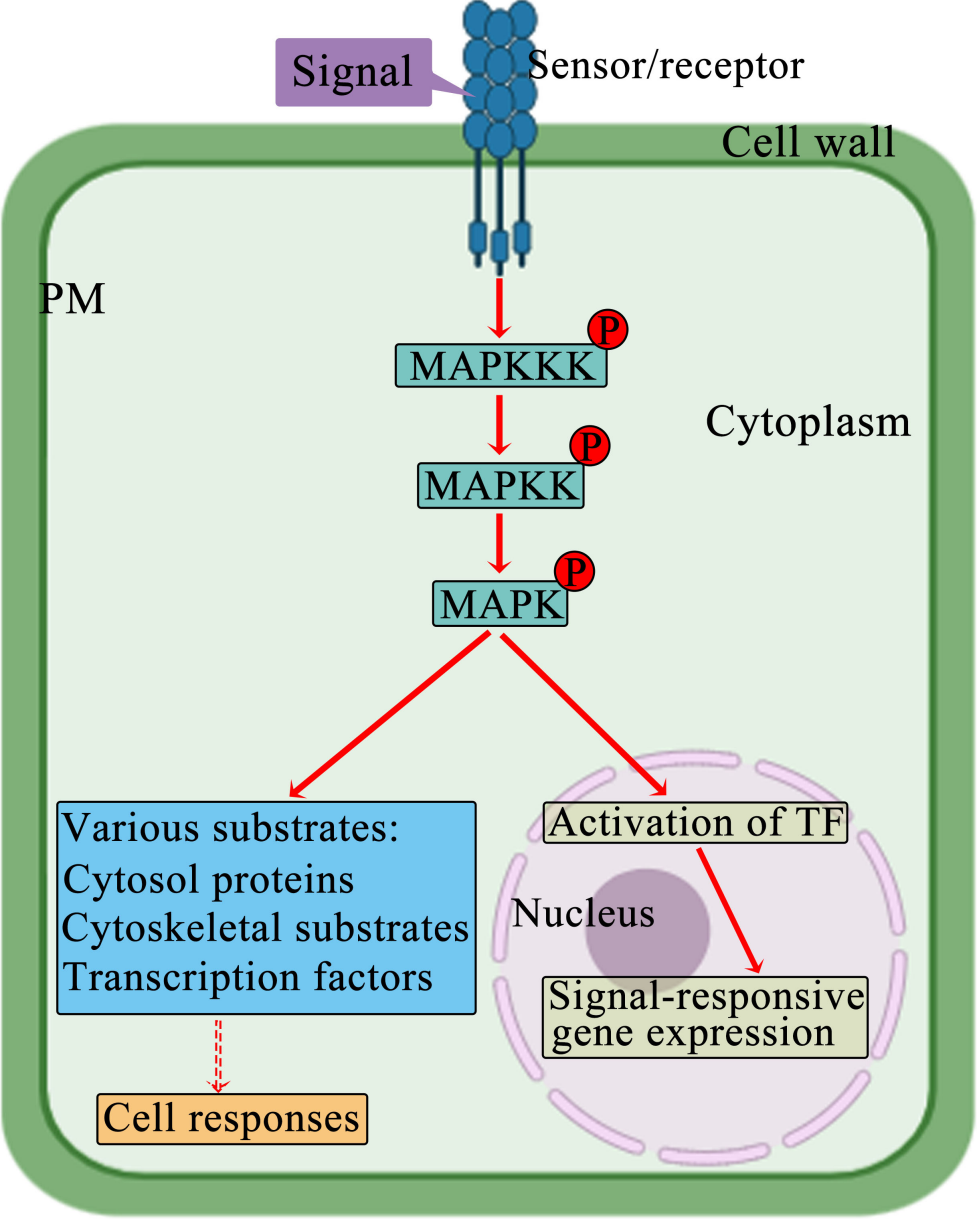
Schematic representation of the MAPK cascade. A signal transduction cascade navigates the signal from MAPKKK to MAPK by triggering a series of Thr/Tyrosine (Tyr) and Ser/Thr phosphorylation events. Eventually, the activated MAPKs are transported to the nucleus, where they phosphorylate transcription factors, altering their binding affinity to the promoter regions of target genes and thereby suppressing or promoting gene expression. The MAPK pathways function as kinases and convert external signals from the environment for modifying specific target proteins, for example, transcription factors, through post-translational processes. This induces reconfiguration of gene expression patterns and an adjustment in the stress response mechanism of the plant.

The MAPK signaling pathway functions as a universally preserved mechanism in eukaryotic organisms for transmitting signals from the cell exterior (Huang et al., 2022). The MAPK cascade participates in plants’ response to manage abiotic stress (de Zelicourt et al., 2016). MAPKs are structurally highly conserved serine/threonine protein kinases; they phosphorylate diverse substrates, for example, cytoskeleton-related proteins, protein kinases, and transcription factors; moreover, they have key functions in controlling how plants respond to stress, such as drought, heavy metals, extreme temperatures, and salinity (Moustafa et al., 2014; Gao et al., 2019). MAPK, MAPKK, and MAPKKK are the three distinct sets of protein kinases in the MAPK cascade. These kinases activate one another sequentially through phosphorylation (Danquah et al., 2014). Typically, MAP3Ks are activated through extracellular signals. The activated MAP3Ks then induce phosphorylation and activation of the S/T-X_3-5_-S/T motif present in downstream MAPKKs. Next, the activated MAPKKs stimulate the phosphorylation and activation of MAPKs at the TXY activation loop, thereby facilitating signal transmission to the nucleus (**Figure 1**) (Tena et al., 2001; Meng and Zhang, 2013; Jiang et al., 2022). MAPKKKs, may serve as adaptors to establish a link between upstream signaling events and the main MAPK cascades.

### 2.2 Phylogeny and classification of MAPKs

**Figure 2:**
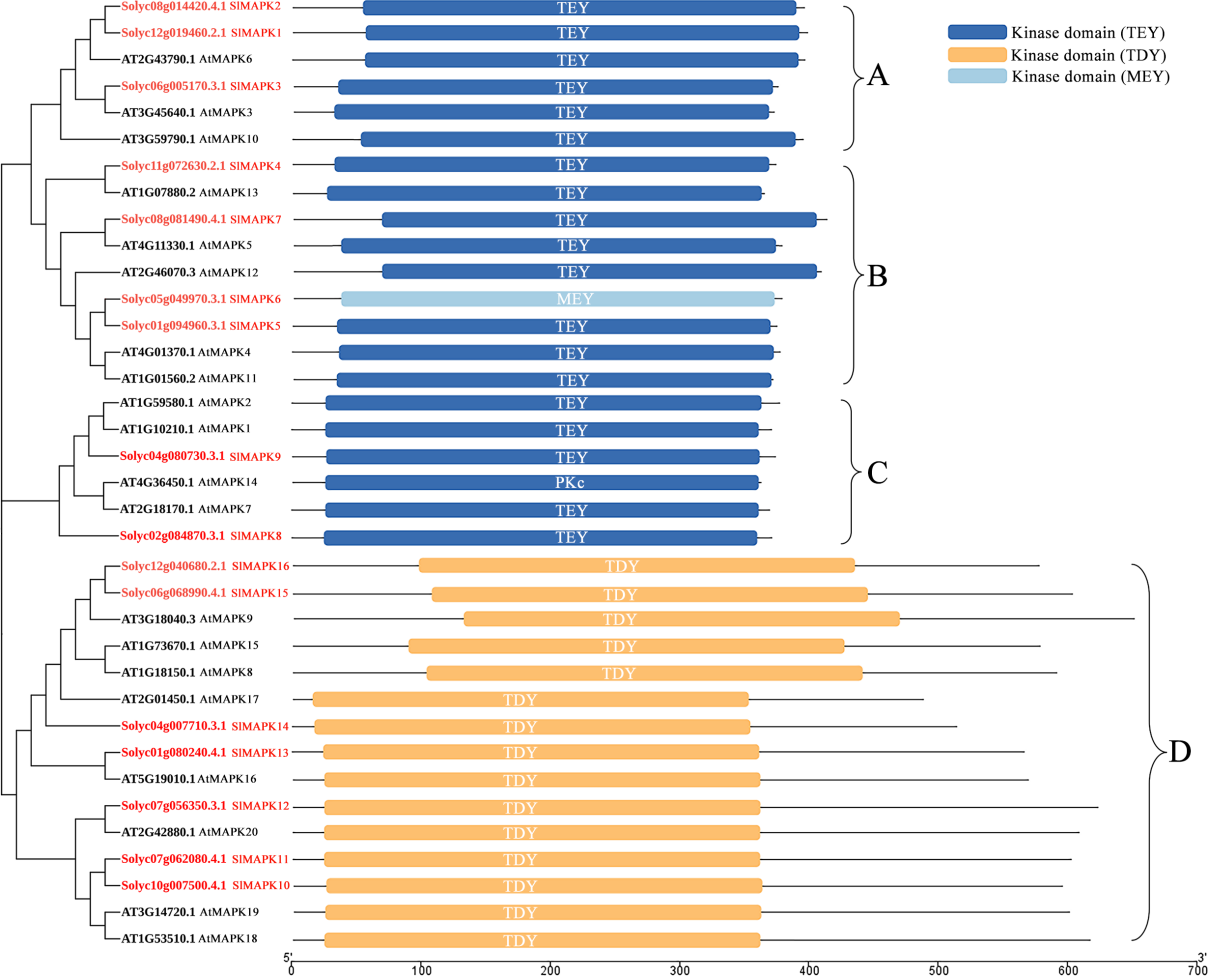
The phylogenetic tree and domain structure of plant MAPKs. On the left, the phylogenetic relationships of MAPKs from tomato (*Solanum lycopersicum*, Sl) and *Arabidopsis thaliana* (At). On the right, the domain structure of the MAPKs. TEY, ZThr-Glu-Tyr; TDY, Thr-Asp-Tyr; MEY, Met-Glu-Tyr.

The complete *Arabidopsis thaliana* genome sequence revealed 20 genes potentially encoding MAPKs. In tomato, 16 SlMAPKs have been identified. These MAPKs are divided into four groups (**Figure 2**) (MAPK Group, 2002; Kong et al., 2012). SlMAPK1-3, SlMAPK4-7, SlMAPK8 and 9, and SlMAPK10-16 were included in Groups A, B, C, and D, respectively. Conserved domain analysis shows that members of the A, B, and C subfamilies have a Thr-Glu-Tyr (TEY) phosphorylation motif in their active sites, while members of the D subfamily have a Thr-Asp-Tyr (TDY) motif in their active sites (Kong et al., 2012). MAPKs of Group A, specifically *Arabidopsis thaliana* MAPK3 and MAPK6, were initially associated with plant defense mechanisms and responses to abiotic stresses. Subsequently, their significant contributions to plant development and growth were also demonstrated (Wang et al., 2023; Wu et al., 2024; Wu and Wang, 2024).

Group B MAPKs, such as *Arabidopsis thaliana* MAPK4, MAPK5, MAPK11, MAPK12, and MAPK13, play roles in plant immune responses, adaptation to environmental conditions, and plant development and growth (de Zelicourt et al., 2016; Thulasi Devendrakumar et al., 2018). Furthermore, Group C MAPKs, including *Arabidopsis thaliana* MAPK1, MAPK2, MAPK7, and MAPK14, along with MAP3K17/18, constitute the MAP3K17/18-MKK3-MAPK1/2/7/14 cascade; these MAPKs have important functions in abscisic acid (ABA) signaling, senescence, and tolerance to drought (Matsuoka et al., 2015; Li et al., 2017; Tajdel-Zielińska et al., 2024). Groups A-C included the TEY subtype MAPKs, while Group D MAPK is a TDY subtype with eight members in *Arabidopsis thaliana*: MAPK8, MAPK9, MAPK15, MAPK16, MAPK17, MAPK18, MAPK19, and MAPK20. Compared to the members of Group A-C, Group D members possess a longer C-terminal region and show absence of the common MAPKK-binding docking motif (Dóczi et al., 2012; Zhang and Zhang, 2022). MAPK9, a Group D member, is activated independent of upstream MAPKKs; it is activated by autophosphorylation. Furthermore, BRASSINOSTEROID-SIGNALING KINASE1 modulates the phosphorylation of MAPK15 to induce resistance in *Arabidopsis thaliana* to powdery mildew (Nagy et al., 2015; Shi et al., 2022). Thus, MAPKs are pivotal in enabling overall development and growth of plants and in interacting with biotic and abiotic stresses.

## 3 Functions of MAPKs in abiotic stresses

**Figure 3:**
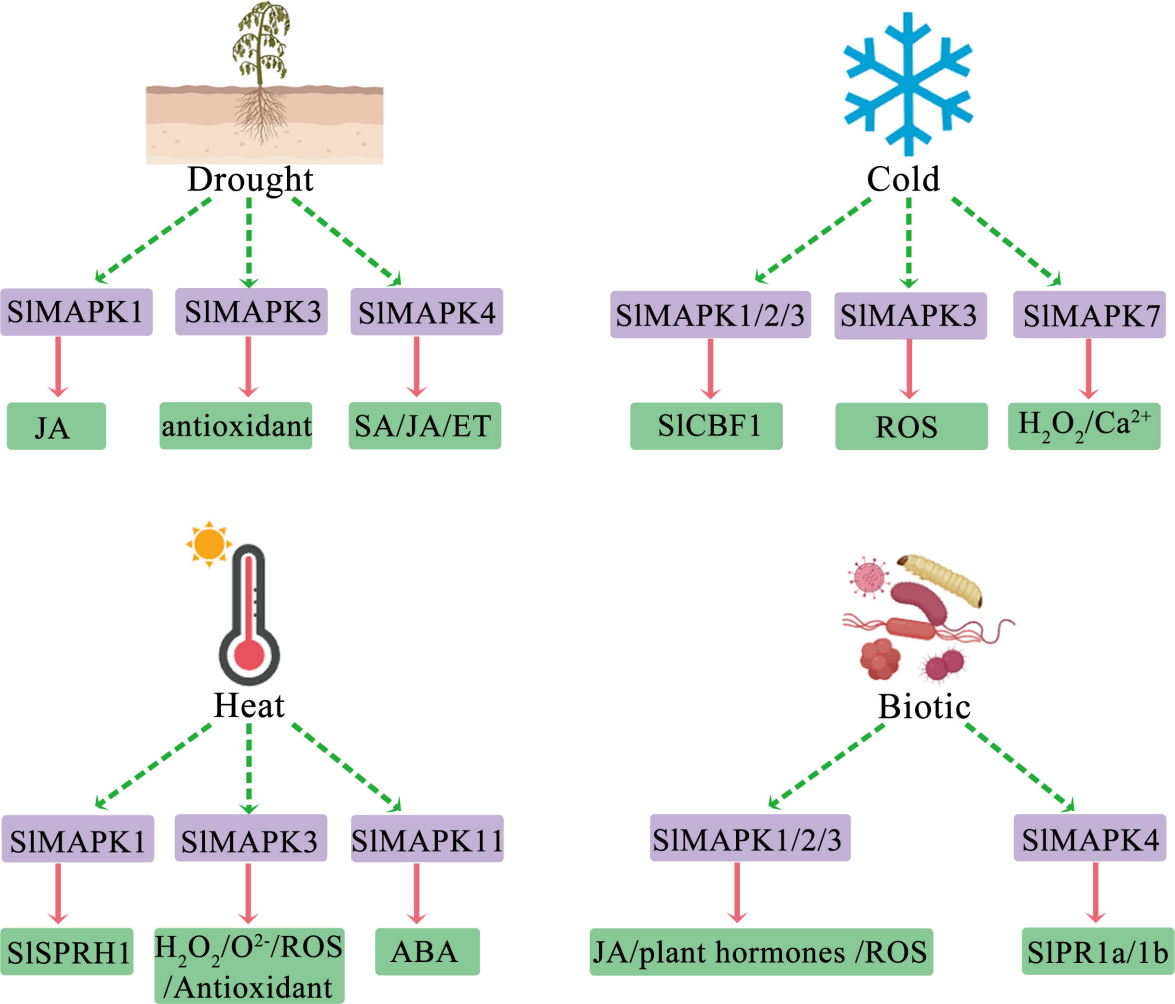
Overview of roles of various SlMAPKs in stress responses and their regulation and potential downstream effects. ET, ethylene; JA, jasmonic acid; SA, salicylic acid; SlCBF1, C-repeat binding factor 1; ROS, reactive oxygen; H_2_O_2_, hydrogen peroxide; SlSPRH1, serine-proline-rich protein homology; ABA, abscisic acid; SlPR1a/b; Pathogenesis-Related 1a/b.

Plants can detect unfavorable environmental changes, including elevated salinity, high temperatures, and drought. They translate these stress indicators into cellular responses, which enable them to modify their development, metabolism, and growth effectively to ensure survival and reproduction (Fujita et al., 2006; Zhu, 2016; Chen et al., 2021). Numerous signaling reactions and events at the physiological, biochemical, and molecular levels are reported in plants experiencing abiotic stresses (Zhang et al., 2022a). Moreover, the MAPK cascade pathway has an essential function in adaptation to various stress events in different plants and at varying development and growth stages (Danquah et al., 2014). MAPK activation serves as a secondary response to physiological alterations that plant cells undergo during stress. Additionally, the absence of sensor mutants complicates the direct association of MAPK activation with specific abiotic stresses (Zhang and Zhang, 2022; Wei et al., 2022). Several plant hormones, such as ABA, jasmonic acid (JA), and ethylene (ET), have crucial functions in the response of plants to abiotic stress (Halim et al., 2006; Lee and Luan, 2012; Gontia-Mishra et al., 2014). Additionally, MAPKs are implicated in the signaling and biosynthesis pathways of these hormones (**Figure 3**).

### 3.1 MAPKs regulate response in tomato plants to drought stress

Drought, one of the major abiotic stress, severely impacts crop production. It not only has drastic effects on plant productivity and metabolism but also leads to widespread socioeconomic burden and damage (Lesk et al., 2016). Several biochemical and transcriptional studies have shown that MAPKs are involved in tomato plant’s response to drought. SlMAPK1 has a pivotal function in how plants respond to abiotic stressors such as drought. As reported previously, SlMAPK1 contributes to JA biosynthesis, a hormone essential for plant defense and stress response (Alfagham et al., 2024). During drought, SlMAPK1 activation increases JA levels, thereby improving the plant’s capacity to withstand dehydration and recuperate from water deficiency. JA is crucial for stomatal closure, which reduces transpiration-mediated loss of water, as well as for root growth that enhances water absorption from soil (Alfagham et al., 2024; Wang et al., 2018). Furthermore, SlMAPK1 interacts with various proteins and transcription factors within the MAPK pathway, thereby activating genes that facilitate plant acclimation to drought stress. Previous research has also indicated that the metabolite albaflavenone, produced by microbial endophytes, improves drought resistance in tomato plants by activating the SlMAPK1 protein in the MAPK signaling pathway (Alfagham et al., 2024). SlMAPK3 has a remarkable function in responding to abiotic stress events, for example, stress due to heavy metals (cadmium) and drought (Muhammad et al., 2019). *SlMAPK3* gene knockdown by CRISPR/Cas9 gene editing technology increased tomato plants’ sensitivity to drought stress, led to greater membrane damage, and reduced antioxidant enzyme activity (Wang et al., 2017a). In contrast, transgenic plants overexpressing SlMAPK3 showed enhanced antioxidant enzyme activity and improved tolerance to stress due to drought and cadmium exposure. These findings revealed the function of SlMAPK3 in regulating antioxidant responses and protecting cellular membrane in tomato plants, thus suggesting potential molecular mechanisms for developing tomato varieties with elevated abiotic stress tolerance (Wang et al., 2017a; Muhammad et al., 2019; Huang et al., 2023). Compared to control plants, *SlMAPK4* gene silencing diminished drought stress tolerance, resulting in earlier wilting in drought (Virk et al., 2013). Additionally, silencing of SlMAPK4 in tomato plants upregulated the expression of Pathogenesis-Related 1a (SlPR1a) and SlPR1b, which are defense-related genes. Thus, in tomato, SlMAPK4 may participate in salicylic acid (SA) - and JA/ET-mediated signaling pathways (Nambeesan et al., 2012; Virk et al., 2013).

Carbon monoxide (CO), nitric oxide (NO), and hydrogen sulfide (H_2_S) are the three endogenously produced gaseous signaling molecules (Yamasaki and Cohen, 2016). Exogenous H_2_S and NO treatment enhances plant stress tolerance against harsh environments, including extreme temperatures, salinity, and drought, by enhancing antioxidant systems and cellular resistance (Tian et al., 2016; Ni et al., 2016; Kolbert, 2019). The MAPK signaling pathway facilitates the promotion of tomato seedling growth by H_2_S, and some MAPK family genes, particularly *SlMAPK3* and *SlMAPK13*, exhibit remarkably altered expression pattern following exogenous addition of NaHS The MAPK inhibitor PD98059 reduces H_2_S levels and L-cysteine demercase (LCD) activity in tomato seedlings (Ba et al., 2021). SNP, an NO donor, at 100 μM concentration effectively promotes tomato seedling growth, enhances nitrogen metabolism, and minimizes oxidative damage in low nitrogen stress condition. The molecular response to low nitrogen concentration stress and NO treatment in tomato seedlings involves the MAPK pathway as a key factor (Zhang et al., 2022b; Lu et al., 2024). PD98059, the MAPK inhibitor, weakens NO effects, thus suggesting that, in low nitrogen concentration stress condition, the MAPK signaling pathway mediates NO-based nitrogen metabolism and abolition of oxidative damage and growth inhibition in tomato seedlings (Ba et al., 2021; Zhang et al., 2022b; Lu et al., 2024).

### 3.2 Response of MAPKs in tomato to temperature stress

Temperature significantly impacts plant productivity and growth. Plants can thrive within a specific temperature range; however, extreme temperatures can cause damage to plant cells. Cold stress inhibits germination, growth, and metabolism and disrupts membranes by forming ice crystals. High temperatures induce heat stress, increase respiration, and cause ROS overproduction, thereby impacting plant productivity and growth (Moustafa et al., 2014; Wu et al., 2022; Manasa et al., 2022; Kan et al., 2023). MAPKs are critically involved in temperature signal perception and transduction (Sangwan and Dhindsa, 2002). MAPKs and C-repeat binding factors (CBF) have essential functions in monitoring cold response. Exogenous hydrogen peroxide treatment improves tomato plants tolerance to cold stress by stimulating SlCBF1 and SlMAPK1/2/3 expression and modulating antioxidant enzyme activity and phytohormone levels (Wang et al., 2017b). *SlMAPK3* knockout resulted in reduced content of ferulic acid (FA) and inhibition of FA synthesis-associated gene expression (*SlC3H*, *SlC4H*, *SlCOMT*, and *SlPAL5*) (Kumar and Pruthi, 2014; Song et al., 2023). Specifically, FA showed reduced effects on osmotic regulatory substances and antioxidant enzymes together with a decline in CBF pathway-associated gene expression. These results revealed that FA positively contributes to the resistance of tomato fruits to chilling stress through MAPK3-dependent upregulation of genes linked with the CBF transcriptional pathway (Miura et al., 2012; Shu et al., 2022). Remarkably, SlMAPK1 and SlMAPK2 are crucial for mediating B-box (SlBBX17) proteins phosphorylation. This phosphorylation event enhances the formation of a complex between SlHY5 and SlBBX17, which then modulates the activity of SlCBFs, ultimately conferring low-temperature tolerance to the plants (Heng et al., 2019; Song et al., 2023).

In tomato plants, the *SlMAPK3* gene participates in environmental stress response, particularly to temperature stress (Yu et al., 2019). *SlMAPK3* gene knockout enhances heat stress tolerance through the prevention of ROS accumulation as well as elevation of the expression levels of heat stress transcription factors (HSFs), antioxidant enzymes, and heat shock proteins (Iba, 2002; Yu et al., 2019). Conversely, *SlMAPK3* overexpression in tobacco enhances low-temperature-stress tolerance by activating cellular antioxidant systems, altering stress-responsive gene transcription, and improving ROS scavenging- and stress tolerance-associated gene expression. Collectively, these findings highlight the critical regulatory function of SlMAPK3 in plants’ response to temperature stress (Yu et al., 2015a, 2019). SlMAPK7 is a Group B MAPK gene. As shown previously, SlMAPK7 responds to various stresses and signaling molecules, and its mRNA expression level is regulated by H_2_O_2_ and calcium ions (Ca^2+^). SlMAPK7 is localized primarily in the cell nucleus, and its overexpression enhances cold stress tolerance in transgenic tomato plants (Yu et al., 2015b). This enhancement is linked to cellular antioxidant system activation, modulation of stress-related gene expression, and more efficient scavenging of ROS. Thus, SlMAPK7 positively regulates plant cell response to cold stress by altering ROS homeostasis and affecting stress-responsive gene expression (Xing et al., 2008; Pitzschke et al., 2009; Yu et al., 2015b). Thus, MAPK signaling is a critical input for plants to recognize and act to tolerate temperature stress and to regulate cold tolerance by activating key pathways and transcription factors, which ultimately enhance growth and productivity under extreme conditions.

The occurrence of extreme weather events has increased recently, with high temperature (HT) being a notable example. Such elevated temperatures have become a primary environmental condition affecting the overall development and growth of crops (Teshome et al., 2020). In tomato plants, heat stress activates MAPKs. Heat-activated MAPK phosphorylates and promotes HSF clade A3 (HsfA3) expression (Link et al., 2002). The increased focus on MAPK-mediated responses to HT is noteworthy. *SlMAPK1* silencing improves the heat tolerance of tomato plants, whereas *SlMAPK1* overexpression leads to a decrease in heat tolerance in transgenic tomatoes. Additionally, following exposure to heat stress, the levels of antioxidant defense proteins in plants with *SlMAPK1* interference are markedly increased, while plants with *SlMAPK1* overexpression show a reduction in antioxidant defense capacity (Ding et al., 2018). SlMAPK1 interacts with the serine-proline-rich protein homology (SlSPRH1) and phosphorylates its Ser-44 site. This interaction regulates the antioxidant defense protein, which participates in response to HT, thus negatively regulating tomatoes’ tolerance to HT (Ding et al., 2018; Mo et al., 2021). CRISPR/Cas9-mediated *slmapk3* mutants exhibited more tolerance to heat stress than WT plants, suggesting that SlMAPK3 was a negative regulator of thermotolerance (Yu et al., 2019). Additionally, the expression of antioxidant enzymes and heat shock proteins/heat shock factors (HSPs/HSFs) genes played a role in the heat stress response mediated by SlMAPK3 in tomato plants (Yu et al., 2019; Mo et al., 2021). SlMAPK11 is a novel gene that influences tomato seed germination, with higher expression in seeds adapted to lower germination temperatures (Song et al., 2021). Overexpression of SlMAPK11 reduces tomato seed germination and increases ABA sensitivity by upregulating 9-cis-epoxycarotenoid dioxygenase (NCED1) and affecting ABA signaling through SNF1-related kinase 2.2 (SnRK2.2) phosphorylation (Lefebvre et al., 2006; Song et al., 2021). Additionally, MAPK11 interacts with SnRK1, potentially inhibiting its activation and influencing ABA-insensitive 5 (ABI5) transcription (Song et al., 2021). Thus, MAPKs have relevant functions in tomato plants in responding to drought and temperature stress, mainly by regulating signaling pathways and antioxidant systems to enhance tomato tolerance.

## 4 Functions of MAPKs in biotic stresses

Stresses induced by attack of pathogens, including bacteria, fungi, viruses, and other microorganisms or macroorganisms, are referred to as biotic stress. These biotic stresses can harm crops and hinder their development and growth at various stages of their lifecycle. Disease is a leading cause of postharvest decay of tomato fruits (Chisholm et al., 2006; Luo et al., 2024). In recent years, various bacterial and fungal phytopathogens have posed a substantial threat to tomato cultivation (Noman et al., 2021). To counter attacks from pathogens, intricate signaling networks have emerged in plants for recognizing these threats and initiating a defensive response (Saijo and Loo, 2020). The MAPK gene family is crucial in responding to pathogen attack and modulates development and growth during different biotic stresses and microbial invasions (**Figure 3**) (Jiang et al., 2022; Majeed et al., 2023).

SlMAPK1, 2, and 3 are activated in terms of MAPK activity in cells cultured in suspension by various stimuli. These stimuli include oligosaccharide elicitors, systemin, Cf-4/Avr4-mediated hypersensitive response (HR), and ultraviolet B (UVB) radiation (Holley et al., 2003; Stulemeijer et al., 2007). Additionally, in tomatoe*s*, MAPK1 and MAPK2 compromise prosystemin-mediated resistance to herbivory by *Manduca sexta* (Lepidoptera), demonstrating that they are also required for successful defense against herbivorous insects (Kandoth et al., 2007). SlMAPK2 and SlMAPK3 play a crucial role in the Pto-mediated defense response in tomatoes. SlMKK4 and SlMKK2 are the upstream MAPKKs that activate these MAPKs and induce cell death, contributing to plant defense signaling (Pedley and Martin, 2004). SlMAPK4 has pivotal functions in inducing resistance to *Botrytis cinerea* infection. Endogenous SlMAPK4 expression knockdown in tomato plants by virus-induced gene silencing (TRV-SlMAPK4) increased the plant’s susceptibility to *B. cinerea*. *SlPR1a* and *SlPR1b*, defense-related genes, showed upregulated expression in *SlMAPK4*-silenced plants. This increased expression might be due to the loss of SlMAPK4 function, which disrupts the SA-mediated signaling pathway (Nambeesan et al., 2012; Virk et al., 2013). SlMKK4 and SlMKK2 can activate SlMAPK1 and SlMAPK2 *in vitro* and enhance resistance to infections caused by *B. cinerea*. Additionally, SlMKK2 and SlMAPK2 show resistance to diseases caused by *Xanthomonas campestris* pv. *vesicatoria* in tomato plants (Melech-Bonfil and Sessa, 2011; Li et al., 2014). Tomato SlMAPK1, SlMAPK2, and SlMAPK3 are activated in response to fungal toxin fusicoccin, and have roles in HR and resistanceD (Higgins et al., 2007; Stulemeijer et al., 2007). SlMAPK1/2/3 inhibition disrupts the defense signaling pathways in tomato fruits and increases susceptibility to infection by *B. cinerea*; plant hormones and ROS are linked with defense signaling pathways related to SlMAPK1/2/3 (Zheng et al., 2015). The dual specificity of SlMAPK3 characterizes a convergence point for numerous signaling pathways that induce defense responses (Mayrose et al., 2004). Two MAPK pathways, MEK1-NTF6 and MEK2-WIPK, are involved in disease resistance mediated by *Pto* in tomatoes through the modulation of NPR1 expression, a crucial regulatory protein for systemic acquired resistance (Ekengren et al., 2003). SlMAPKKKϵ has a pivotal function in HR-stimulated cell death and confers tomato with resistance to gram-negative bacterial infections through the mediation of the SlMAPKKKϵ-MEK2-WIPK/SIPK signaling pathway. SlMAPKKKϵ silencing weakened the defense of tomato plants against *P. syringae* and *X. campestris* strains, leading to the onset of disease symptoms and increased bacterial proliferation (Melech-Bonfil and Sessa, 2010). Thus, MAPK cascades through several signal transduction pathways regulate disease resistance in tomato plants.

## 5 Conclusions and future prospects

MAPKs play a key role in tomato plants response to biotic and abiotic stresses. MAPKs regulate immune responses, overall development, and adaptation to environmental changes in tomatoes through various signaling pathways (Kong et al., 2012; Majeed et al., 2023). In particular, during abiotic stress events, for example, drought, high temperatures, and salinity, specific members of the MAPK family enhance tomato tolerance by modulating plant hormone signaling, activating antioxidant systems, and protecting cell membranes (Wang et al., 2017a). The MAPK cascade also has vital functions in plant defense mechanisms during biotic stress, including resistance to diseases such as gray mold (Meng and Zhang, 2013). Further elucidation of the pertinent functions of the MAPK cascade in the response of tomato plants to stress can clarify the underlying molecular mechanisms that could enable to develop tomato varieties with enhanced stress resistance.

At present, the functions of many protein kinases in the tomato MAPK cascade pathway are not yet fully understood, particularly those of MAPKK and MAPKK-related kinases, which should be further studied in the future. There are numerous members of the plant MAPK cascade protein kinase family, which can combine to form various levels of MAPK pathways to respond quickly to a variety of stressors (MAPK Group, 2002). It is now understood that a stimulus generally requires the participation of multiple cascades (Kumar et al., 2020). Therefore, the interrelationships between members at all levels and how these cascade pathways collaborate to maintain signal specificity in terms of functions and cross-talk still require substantial research to be fully understood. Similarly, since external stimuli are diverse and the MAPK resources of the plant itself are limited, a cascade pathway often plays a role in multiple stimulus responses (Wu and Wang, 2024). In these metabolic networks, they are usually connected in series through phosphorylation (Rodriguez et al., 2010). However, there are relatively few studies on phosphorylation and key phosphorylation sites, and further research is urgently needed.

Future research should explore the crosstalk between the MAPK cascade and other signaling molecules, such as plant hormones and transcription factors, to better understand how these interactions influence tomato adaptability to stress (Amoroso et al., 2023). Additionally, by using gene editing technologies such as CRISPR/Cas9, we can precisely control the expression levels of specific genes linked with the MAPK cascade; this could enable to confirm their functions through molecular analysis and provide candidate genes to develop tomato varieties with enhanced stress resistance. The development and application of high-throughput screening technologies for identifying and validating new MAPK cascade members and their interacting proteins should also be focused. This could facilitate better understanding of the MAPK signaling network and provide molecular tools for designing new crop improvement strategies. Finally, with the increasingly severe impact of global climatic changes on agricultural production, studies on the influence of the MAPK cascade on tomato stress responses are not only scientifically significant but also practically valuable. As the signal transduction pathways and mechanisms mediated by MAPK under stress are revealed, this can provide a theoretical basis for tomato stress resistance breeding.
